# Improved Reconstruction of Chaotic Signals from Ordinal Networks

**DOI:** 10.3390/e27050499

**Published:** 2025-05-06

**Authors:** Antonio Politi, Leonardo Ricci

**Affiliations:** 1 Department of Physics, University of Aberdeen, Aberdeen AB24 3UE, UK; 2 Institute for Complex Systems, National Research Council, (ISC-CNR), Via Madonna del Piano 10, 50019 Sesto Fiorentino, Italy; 3 Department of Physics, University of Trento, 38123 Trento, Italy; 4 Center for Mind/Brain Sciences, CIMeC, University of Trento, 38068 Rovereto, Italy

**Keywords:** permutation entropy, time series analysis, ordinal patterns, noise

## Abstract

Permutation entropy is customarily implemented to quantify the intrinsic indeterminacy of complex time series, under the assumption that determinism manifests itself by lowering the (permutation) entropy of the resulting symbolic sequence. We expect this to be roughly true, but, in general, it is not clear to what extent a given ordinal pattern indeed provides a faithful reconstruction of the original signal. Here, we address this question by attempting the reconstruction of the original time series by invoking an ergodic Markov approximation of the symbolic dynamics, thereby inverting the encoding procedure. Using the Hénon map as a testbed, we show that a meaningful reconstruction can also be made in the presence of a small observational noise.

## 1. Introduction

In our epoch, huge amounts of data are continuously stored and processed to extract relevant information. Artificial intelligence [1] is increasingly being used with this goal in mind, but the results, although helpful in the short term, are opaque and do not directly provide insight on why given phenomena are actually occurring. Here, we address this problem in the context of time series [2], a subject of crucial relevance in many scientific areas: from medical signals (ECG, EEG, MEG), to the functioning of mechanical devices, weather forecasts, and so on [3].

The starting point is the representation of a time series as a sequence of ordinal patterns [4], built without paying attention to the actual values of the recorded variable, but taking into account only the mutual ordering. This is the core of the approach introduced by Bandt and Pompe [5], who proposed quantifying the complexity of a signal in terms of the so-called permutation entropy (PE). The first step consists of building a sequence of *m*-dimensional vectors $X_{n}$ out of a given scalar time series $\left\{x_{n}\right\}$ by using the embedding approach proposed long ago by Takens [6]. In the ordinal pattern context, Cao et al. [7] proposed to build the vectors by assembling equispaced sampled variables, $X_{n}\equiv \left\{x_{n},\;x_{n+L},\ldots ,\;x_{n+(m-1)L}\right\}$, where the lag *L* is any integer ⩾ 1. The vector $X_{n}$ is then encoded as a symbolic sequence (permutation) $W_{n}=\left\{w_{n}^{1},\;w_{n}^{2},\ldots ,\;w_{n}^{m}\right\}$, henceforth also referred to as a word, where the integer $w_{n}^{j}$ belongs, like the index *j*, to the range [1,m] and corresponds to the rank, from the smallest to the largest, of $x_{n+(j-1)L}$ within $X_{n}$.

As a result, the initial time series is transformed into a sequence of words. The corresponding PE, evaluated as the Shannon entropy [8] of the word frequencies, is typically used as an indicator of the underlying unpredictability. The resulting indicator has been implemented to address many different classes of signals and classify their complexity [9], reveal changes in the dynamics [7], identify elements of determinism [10,11,12].

Interestingly, it has been shown that for large enough partition order *m*, i.e., the length of the windows used for the encoding, the PE is a proxy of the Kolmogorov–Sinai entropy, though typically being an overestimate thereof [13]. A strength of the method is that it does not require an explicit partitioning of the phase space, a task that is indeed unavoidably system-dependent and thus represents an additional burden. However, the implicit partitioning induced by the grouping of trajectories in different ordinal patterns also represents a weakness, since it washes out possibly relevant differences between equally coded trajectories. For a not-too-large *m*, it might happen that hidden deterministic degrees of freedom are not resolved, or that a correlated noise induces pseudo-deterministic restrictions to the multiplicity of observable sequences. In order to shed light on those controversial effects, here we propose to complement the symbolic encoding with an inverse procedure to reconstruct a time series akin to the original one. The reliability of the method will directly confirm or disprove the correctness of the initial encoding.

The approach described and implemented in this paper is an elaboration of the method proposed by Small [14] and further explored in [15,16]. A first difference is in the procedure we propose for the construction of a recurrent network that is as insensitive as possible to the presence of observational noise. The underlying idea is a careful selection of words whose probability can be determined in a statistically reliable way. This approach, implemented for the Hénon map [17], can be seen as a process of hierarchical clustering [18].

A second difference is the way time series are eventually reconstructed: suitably attributing weights to the words leads to a more accurate reconstruction. As a result, a simple protocol is defined, based on a Markov approximation. While we do not argue that this approach should replace standard methods like spectral analysis, or the identification of suitable sets of ordinary differential equations, we claim it to be a powerful tool that can profitably complement the existing methods.

The paper is organized as follows. The direct encoding in terms of symbolic sequences as well as the generation of an approximating Markov model is discussed in Section 2. The inverse procedure, namely the reconstruction of the deterministic dynamics out of the Markov model, is the topic of Section 3. Section 4 describes the application of the method to the prototypical example of a time series generated by the Hénon map, also when it is contaminated by observational noise. Remaining open problems are outlined in the final Section 5.

## 2. Direct Encoding

As mentioned above, the lag *L* can, in general, be any strictly positive integer number, though it is surely inconvenient to assume it so large that the resulting words would be essentially indistinguishable from a random process (in the presence of chaos), a phenomenon also known as irrelevance. Here, since we deal with a discrete-time map, we set L=1, a choice able to capture the nonlinearities, while minimizing the folding effects that tend to hide the underlying determinism.

Setting the partition order to a value *m*, and assuming the input time series, or signal, to be sufficiently long, we identify all observed words and their probabilities from the respective frequencies along the signal itself. The words are then ranked according to their probability. Let $P_{k}$ denote the probability of the *k*-th most probable word $W^{k}$, i.e., $P_{k}\propto \#\left\{n|W_{n}=W^{k}\right\}$. The words can be interpreted as the nodes of a network, and the original signal as a path on the network itself [14]. The directed connections among the nodes are the transitions $W^{k}\to W^{i}$, also known as edges or links, observed while scanning the time series. Even in the most stochastic system, there are at most *m* different links departing from a given node $W^{k}$. This limit to diversity comes from the obvious requirement that, in the trajectory encoded as $W^{k}$, the last (m−1) values are, by construction, the same as the first (m−1) values of the next trajectory, encoded as $W^{i}$; their mutual ordering must be the same. Additionally, determinism and correlations further reduce the number of actual connections.

Irrespective of whether the original system is deterministic—and possibly chaotic—or stochastic, the path on the network is probabilistic. Our next goal is to generate sequences akin to the original one, under the approximation of a Markov process of unitary memory, i.e., a Markov chain. More accurate representations could be implemented, but since we want to test the meaningfulness of our whole approach, we define a procedure that is as simple as reasonably possible.

In order to produce a Markov process, it is necessary (and sufficient) to determine the rate $Q_{ik}$ of the transition from each $W^{k}$ to each $W^{i}$. The next step consists of simplifying the model by removing effectively improbable and therefore negligible transitions. The core motivation of this approach is the development of a reconstruction procedure that might also work in the presence of a weak observational noise (see, for example, [19]), which leads to the appearance of several spurious low-probability nodes.

Deciding that the irrelevant nodes are those whose probability is smaller than some threshold is too naive an idea, since the supposedly relevant nodes are not necessarily arranged in a recurrent network. Hence, we propose to start ordering the $Q_{ik}$ transition frequencies, from the most to the least populated one, then scanning the list, and progressively including the items into a new list until the transitions produce a recurrent network that contains a single ergodic component. More precisely, since each transition is, by definition, a transition from a starting (*S*) to an arrival (*A*) node, two separate catalogs of *S* and *A* nodes are built; the procedure goes on until these two lists coincide. In fact, a minimal necessary condition for a network to be recurrent is that each node is both a potential starting and an arrival point of a network path. Since this condition is not sufficient to ensure the presence of a single ergodic component, it is necessary to check whether this is true; in all simulations, we have not found a single exception. In the end, we are at least sure that the associated stochastic process is well defined.

As a last step in the construction of a minimal Markov model, we renormalize the probabilities $P_{k}$ and the frequencies $Q_{ik}$ upon removing the excluded ones; henceforth, for the sake of simplicity, we keep the same notations. The transition probabilities can thus be estimated as

$$P_{i|k}=\frac{Q_{ik}}{P_{k}}\;.$$

Let μ be the number of nodes that make up the recurrent network, and *M* the μ×μ stochastic matrix describing it: $M_{ik}=P_{i|k}$. It is also convenient to define as ν the number of links, which corresponds to the number of nonzero elements of *M*.

## 3. Inverse Procedure

As already mentioned, even in low-dimensional chaos, the symbolic representation is an intrinsically stochastic process. The question is to what extent is it possible to retrieve the information content of the initial signal, thereby revealing its deterministic nature, if any. Pursuing this goal, we henceforth consider a sequence $\left\{{\hat{W}}_{n}\right\}$ of words randomly generated according to the approximating Markov process defined above: for any *n*, ${\hat{W}}_{n}=W^{k}$, where the index *k* takes on an integer value between 1 and μ.

In the direct encoding, the same word $W^{k}$ is shared by trajectories exhibiting the same temporal pattern, regardless of the actual values of the recorded variable. Let $S(W^{k})$ be the set of all trajectories that are encoded as $W^{k}$: $S\left(W^{k}\right)=\left\{X_{n}|W_{n}=W^{k}\right\}$. Whenever the dispersion within $S(W^{k})$, i.e., the fluctuations among the components of the trajectories encoded by the same word $W^{k}$, is sufficiently small, it looks plausible that a faithful copy of the original signal can be reproduced.

Let $\bar{X}\left(W^{k}\right)=\left\{{\bar{X}}_{1}\left(W^{k}\right),{\bar{X}}_{2}\left(W^{k}\right),\ldots ,{\bar{X}}_{m}\left(W^{k}\right)\right\}$, where, for each j∈[1,m], ${\bar{X}}_{j}\left(W^{k}\right)\equiv {\langle x_{n+j-1}\rangle }_{n}$, represent the average sample trajectory encoded by the word $W^{k}$. Similarly, let $\sigma_{j}\left(W^{k}\right)$ denote the standard deviation of the *j*-th component of the average trajectory.

**Figure 1:**
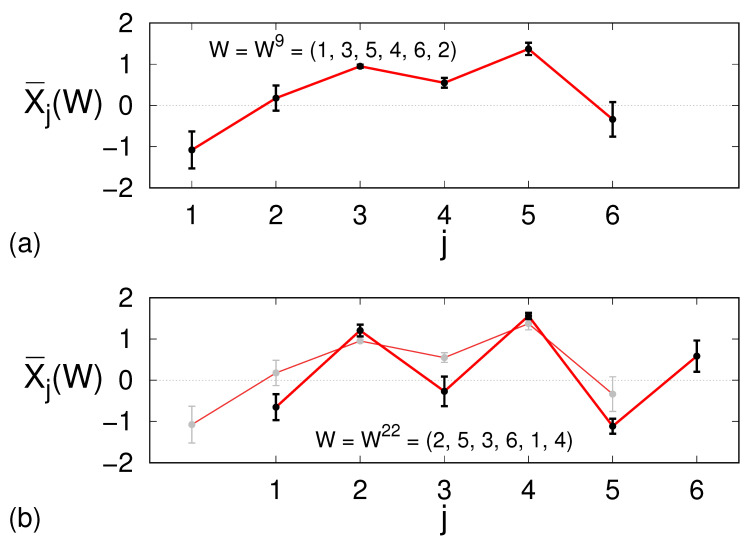
(**a**) Average trajectory corresponding to the word $W^{9}=\left(1,\;3,\;5,\;4,\;6,\;2\right)$, which is ranked 9-th in the m=6 encoding of the Hénon map. The dots denote the average values at each time instant, while the error bars correspond to the standard deviations. (**b**) Average trajectory corresponding to the word $W^{22}=\left(2,\;5,\;3,\;6,\;1,\;4\right)$, namely one of the three that, in the m=6 encoding of the Hénon map, follow the former word $W^{9}=\left(1,\;3,\;5,\;4,\;6,\;2\right)$. This last word is once more reproduced in the background for comparison. For each shared point of the two trajectories, it is apparent how the average values and the respective standard deviations differ from one word to the other one.

In ref. [13], it was found that the $\sigma_{j}$’s progressively decrease upon increasing the length *m*; this is an indirect indication that the encoding provides an asymptotically unique representation of the dynamics, or equivalently that the underlying partition of the phase space is a generating one. However, the decrease in $\sigma_{j}$ upon increasing *m* is true also for white-noise signals (once a set of *m* randomly generated numbers are ranked, the fluctuation of the actual value of the set’s *k*-th element decreases as *m* is increased). Hence, it is not a priori obvious whether a given encoding can provide a sufficiently accurate description of the time series. In Figure 1a, for the Hénon map

(1) $$x_{n+1}=a-x_{n}^{2}+bx_{n-1}\;,$$

with a=1.4 and b=0.3, we show an instance of an average trajectory with m=6, namely the one corresponding to the word $W^{9}=\left(1,\;3,\;5,\;4,\;6,\;2\right)$, as well as the relative spreading.

The relatively small σ values encourage us to interpret, for example, ${\bar{X}}_{1}\left({\hat{W}}_{n}\right)$ as the value $y_{n}$ of a synthetic time series to be associated to the word $W_{n}$. There is, however, a problem. As apparent in Figure 1b, where the average trajectories $\bar{X}$ are displayed for two consecutive words, we can see that the last five values of the first averaged trajectory do not coincide with the first five of the following one. A reasonable way to cope with this uncertainty is to define the synthetic value $y_{n}$ as the average over all possible expected values as

$$y_{n}=\frac{1}{m}\sum_{j=0}^{m-1}{\bar{X}}_{m-j}\left({\hat{W}}_{n-m+j+1}\right)\;.$$

Furthermore, mimicking the evaluation of the sample mean of a non-homoscedastic set of samples, an improved reconstruction rule reads

(2) $$y_{n}=\frac{\sum_{j=0}^{m-1}\frac{{\bar{X}}_{m-j}\left({\hat{W}}_{n-m+j+1}\right)}{\sigma_{m-j}^{\alpha }\left({\hat{W}}_{n-m+j+1}\right)}}{\sum_{j=0}^{m-1}\frac{1}{\sigma_{m-j}^{\alpha }\left({\hat{W}}_{n-m+j+1}\right)}}\;,$$

where the previous rule corresponds to α=0. In this last expression, one would set α=2 if the samples were normally distributed. As this is typically not the case for the situation at hand, we opted for the less strict value α=1. Some tests (see the discussion below on the PE values) showed that the use of the reciprocal standard deviations (α=1) instead of the reciprocal variances (α=2) as weights indeed provides a good compromise.

Finally, it is important to note that, once the Markov transition matrix *M* is given, the proposed decoding procedure unavoidably yields a finite number $N_{p}\left(m\right)$ of points in the original phase space. Provided that all terms of the sum in the numerator of Equation (2) are different from one another, the “density” $N_{p}\left(m\right)$ turns out to be equal to the number of possible *m*-tuples $[{\bar{X}}_{m}\left({\hat{W}}_{n-m+1}\right)$, ${\bar{X}}_{m-1}\left({\hat{W}}_{n-m+2}\right)$, …, ${\bar{X}}_{1}\left({\hat{W}}_{n}\right)]$ that are generated by the Markov process (here *n* can be any number), or equivalently, the number of possible *m*-tuples $\left(j_{1},j_{2},\ldots ,j_{m}\right)$, where $1⩽j_{i}⩽\mu$, ∀i.

Altogether, $N_{p}\left(m\right)$ can be obtained as follows. Let H be a Boolean matrix whose elements $H_{ik}$ are equal to 1 if the transition k→i exists, or equal to 0 if it is forbidden. If m=2, $N_{p}\left(2\right)=\sum_{ik}H=\nu$. Considering m⩾3, an *m*-tuple $\left(j_{1},j_{2},\ldots ,j_{m}\right)$ exists if

$$H_{j_{1},j_{2}}\cdot H_{j_{2},j_{3}}\cdot \ldots H_{j_{m-1},j_{m}}=1\;.$$

It is then straightforward to show that

$$N_{p}\left(m\right)=\sum_{i=1}^{\mu }\sum_{k=1}^{\mu }{\left[H^{m-1}\right]}_{ik}\;.$$

We expect that, the larger the *m*, the more densely the $N_{p}\left(m\right)$ points fill the phase space (see, for example, the values reported in the rightmost column of Table 1 below).

## 4. A Prototypical Example: Hénon Map

To test the performance of the method, we applied it to a time series of $10^{8}$ points generated by the Hénon map described by Equation (1) and using three different values of *m*: m=6, m=8, m=10. In addition, we analyzed, with m=6, the Hénon map perturbed by observational noise: a perturbed time series is obtained by adding to each value $x_{n}$ a realization of a continuous random variable uniformly distributed in the range $\left[-\sigma \sqrt{3},\sigma \sqrt{3}\right]$, thus having a standard deviation σ.

**Figure 2:**
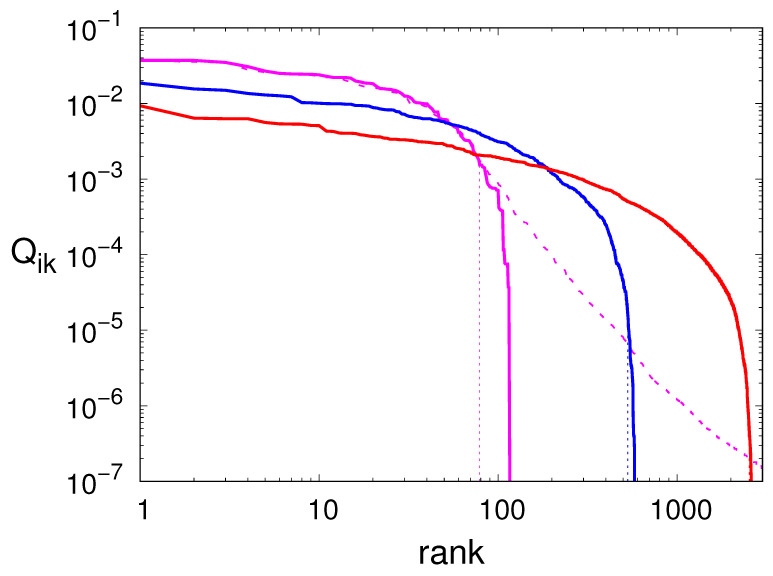
Frequencies $Q_{ik}$ of all transitions observed in a sequence of $10^{8}$ points generated by the Hénon map upon encoding it with words of length m=6 (magenta), m=8 (blue), m=10 (red). The dashed line corresponds to the m=6 encoding acting on the time series perturbed by an observational noise of σ=0.05. For each order *m*, and indeed not visible for m=10, the vertical dotted line represents the number ν of links used to set up the Markov approximation.

In Figure 2, we plot the frequencies $Q_{ik}$ of all transitions for the deterministic model, ranked in decreasing order (see the solid curves). The vertical logarithmic scale reveals a large dispersion of the actual values, which cover several decades. This upholds the argument that it should be legitimate to neglect the least probable transitions. The horizontal logarithmic scale, instead, reveals an exponential growth with *m* of the number of transitions: a manifestation of the topological entropy of this dynamical system.

The procedure discussed in the previous section shows that, in the case m=6, the smallest recurrent network involves μ=51 nodes out of 65 observed while scanning a time series of $10^{8}$ points, and ν=79 transitions out of 120 observed ones (see Table 1). The vertical dotted line in Figure 2 shows the location of the critical point which represents the border of the interval of “acceptable” transitions. The fraction of discarded transitions is therefore about 21%, whereas the discarded mass is just 2.5%: a very small number indeed. The reported numbers reveal the sparsity of the stochastic matrices used in the Markov approximations. Notice also that the fraction of nodes used for the reconstruction of the signal tends to grow for increasing *m*, indicating that more of them become essential.

**Figure 3:**
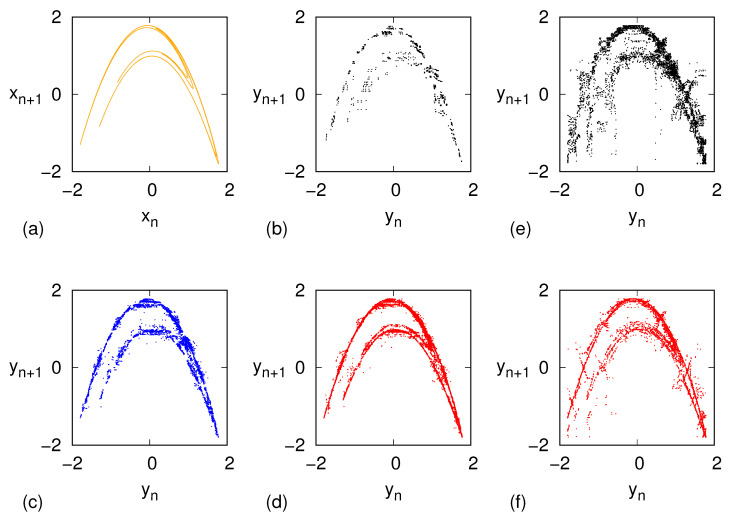
(**a**) Phase portrait of the Hénon map (orange dots), where $v_{n}=x_{n}$. (**b**–**d**) Reconstructed evolution via ordinal pattern encoding and Markov model, according the protocol described in the present work, with depth m=6 ((**b**), black dots), m=8 ((**c**), blue dots), m=10 ((**d**), red dots). (**e**,**f**) Reconstructed evolution via ordinal pattern encoding and Markov model, following the protocol described in [15] with depth m=6 ((**e**), black dots), m=10 ((**f**), red dots).

Next, we let the associated Markov process evolve so as to generate a sequence $\left\{{\hat{W}}_{n}\right\}$ of synthetic words, which are afterwards decoded to generate the corresponding $y_{n}$ sequence according to Equation (2). Figure 3 shows the phase-space evolution of the original model (panel a) along with the ones resulting from our inverse procedure for the three values of *m* considered (panels b–d).

The large-scale deterministic character of the model is already captured for the lowest depth m=6, where the finiteness of the points is clearly visible as $N_{p}\left(6\right)=658$. However, upon increasing *m*, $N_{p}\left(m\right)$ increases rapidly and tinier details are progressively resolved, as revealed by panels c,d in Figure 3. Remarkably, the first level of the fractal structure of the Hénon map is clearly visible for m=10.

For the sake of comparison, Figure 3 also shows the results of a reconstruction procedure akin to the one used in ref. [15], for the cases m=6 (panel e) and m=10 (panel f) as follows. Let $W^{k}$ be the node visited at the *n*-th step of the random walk generated by the stochastic matrix *M*, i.e., ${\hat{W}}_{n}=W^{k}$. Then $y_{n}$ is set as the first element of a randomly chosen trajectory, with replacement, among those that belong to $S(W^{k})$. The improvement of the present approach is visually apparent.

More quantitatively, Table 2 reports the values of the PE computed, for each of the three *m* values, on the pristine Hénon time series as well as on time series generated via the two reconstruction methods, namely the present approach and the protocol, described above, modeled on ref. [15]. The values produced by the present approach are very close to the original ones, while the older protocol yields significantly larger PE values. The reason is that the generation of a “new" *x* value as the average of the last component over all possible *m*-tuples encoded by the same word preserves the mutual ordering (among the last *m* components), while this is not guaranteed if the new variable is selected randomly among all possible values.

Finally, we analyze the noise-perturbed case with m=6. The dashed line in Figure 2 shows that many more links appear in the presence of an observational noise characterized by σ=0.05. Actually, their number is more than 50 times larger than in the deterministic case (see Table 1). In spite of this huge variation, the Markov network obtained by implementing the method proposed in the previous section contains exactly the same number of nodes and links (see again Table 1). The efficacy of the “filtering effect” is confirmed by Table 2 where we see that, while the PE of the noisy signal is significantly larger than that of the deterministic one, the PE of the Markov model is in line with the value of the pristine time series.

**Figure 4:**
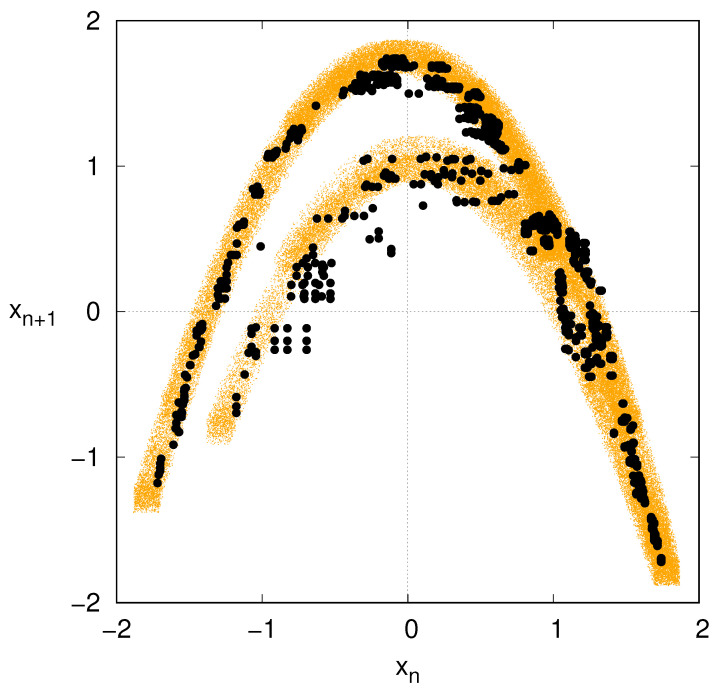
Phase portrait of the Hénon map perturbed by an observational noise of σ=0.05 (orange dots). Reconstructed evolution via ordinal pattern encoding and Markov model with depth m=6 (black dots).

A last comparison is made in Figure 4, which displays the phase portrait of the noise-perturbed Hénon map, and the related reconstruction via Markov approximation with m=6. While the customary Hénon map profile is blurred by the presence of the additive, observational noise, the reconstructed attractor is more defined, indeed resembling the noiseless case of Figure 3b.

The Markov approximation therefore seems to act as a nonlinear filter that enables us to reconstruct the noiseless dynamics, at least as far as the noise is sufficiently small.

## 5. Conclusions and Open Issues

In this paper, we have revisited the method proposed in ref. [15], for the reconstruction of irregular signals, with the goal of making it more robust to the presence of observational noise. Although the outlined application to the Hénon map is very promising, several issues should be further tested. In this paper, having studied a discrete-time map, we have everywhere assumed L=1, but if one decided to deal with continuous-time signals, it might be more convenient to consider a short sampling time accompanied by a larger *L* value. This way, one could better reproduce the continuity of the original signal, although difficulties may be expected in the reconstruction of the underlying Markov approximation (see, e.g., the necessity of implementing algorithms to check for constrained random walks [15]).

Another question is the choice of the window length *m*. In a strictly deterministic system, it is, in principle, convenient to increase *m* as much as possible, since the size of the “cells” that encode the different words progressively decreases. A limitation is imposed only by the computational load. However, in the presence of noise, a cell size smaller than the noise amplitude does not imply an improved accuracy. In practice, it does not make sense to consider windows that are too long. How can an optimal *m* value be identified? Possibly useful information might come from the principal component analysis [22], which can provide a more accurate identification of the cell size, by distinguishing the uncertainty along different directions. Additional help can come from the implementation of appropriate indicators quantifying the degree of predictability (see, for example, ref. [23]).

## Figures and Tables

**Table 1:** Statistics of the Markov approximation for the Hénon map. The row labeled with $6^{★}$ refers to the Hénon map perturbed by an observational noise of σ=0.05.

	# of Available		# of Network’s		Discarded	Phase-Space
m	Nodes	Links	Nodes, *μ*	Links, *ν*	Mass Ratio	Density, $N_{p}\left(m\right)$
6	65	120	51	79	$2.5\cdot 10^{-2}$	658
$6^{★}$	720	4318	51	79	$6.6\cdot 10^{-2}$	658
8	299	590	277	530	$2.5\cdot 10^{-4}$	54,903
10	1406	2680	1344	2530	$2.2\cdot 10^{-5}$	875,390

**Table 2:** PE computed on segments of $10^{5}$ points of the Hénon map and the Markov approximations for m=6,8,10. The $6^{★}$ row refers to the Hénon map perturbed by an observational noise of σ=0.05. The number of significant digits reported is consistent with the expected uncertainty for PE being evaluated out of a time series [20,21].

m	Hénon Map	Present Work’s Markov Approximation	Reconstruction According to [15]
6	3.66	3.63	4.21
$6^{★}$	3.81	3.63	-
8	4.96	4.93	5.68
10	6.31	6.26	6.80

## Data Availability

No new data were created or analyzed in this study. Data sharing is not applicable to this article.
